# The Initial Human Atherosclerotic Lesion and Lipoprotein Modification—A Deep Connection

**DOI:** 10.3390/ijms222111488

**Published:** 2021-10-25

**Authors:** Michael Torzewski

**Affiliations:** Department of Laboratory Medicine and Hospital Hygiene, Robert Bosch-Hospital, 70376 Stuttgart, Germany; michael.torzewski@rbk.de; Tel.: +49-711-8101-3500

**Keywords:** initial atherosclerotic lesion, lipoprotein insudation, enzymatically modified LDL (eLDL), oxidized LDL (oxLDL), macrophage, C-reactive protein, complement system

## Abstract

Atherosclerosis research typically focuses on the evolution of intermediate or advanced atherosclerotic lesions rather than on prelesional stages of atherogenesis. Yet these early events may provide decisive leads on the triggers of the pathologic process, before lesions become clinically overt. Thereby, it is mandatory to consider extracellular lipoprotein deposition at this stage as the prerequisite of foam cell formation leading to a remarkable accumulation of LDL (Low Density Lipoproteins). As progression of atherosclerosis displays the characteristic features of a chronic inflammatory process on the one hand and native LDL lacks inflammatory properties on the other hand, the lipoprotein must undergo biochemical modification to become atherogenic. During the last 25 years, evidence was accumulated in support of a different concept on atherogenesis proposing that modification of native LDL occurs through the action of ubiquitous hydrolytic enzymes (enzymatically modified LDL or eLDL) rather than oxidation and contending that the physiological events leading to macrophage uptake and reverse transport of eLDL first occur without inflammation (initiation with reversion). Preventing or reversing initial atherosclerotic lesions would avoid the later stages and therefore prevent clinical manifestations. This concept is in accordance with the response to retention hypothesis directly supporting the strategy of lowering plasma levels of atherogenic lipoproteins as the most successful therapy for atherosclerosis and its sequelae. Apart from but unquestionable closely related to this concept, there are several other hypotheses on atherosclerotic lesion initiation favoring an initiating role of the immune system (‘vascular-associated lymphoid tissue’ (VALT)), defining foam cell formation as a variant of lysosomal storage disease, relating to the concept of the inflammasome with crystalline cholesterol and/or mitochondrial DAMPs (damage-associated molecular patterns) being mandatory in driving arterial inflammation and, last but not least, pointing to miRNAs (micro RNAs) as pivotal players. However, direct anti-inflammatory therapies may prove successful as adjuvant components but will likely never be used in the absence of strategies to lower plasma levels of atherogenic lipoproteins, the key point of the perception that atherosclerosis is not simply an inevitable result of senescence. In particular, given the importance of chemical modifications for lipoprotein atherogenicity, regulation of the enzymes involved might be a tempting target for pharmacological research.

## 1. Introduction

Over 100 years ago, Klotz and Mannig made the following statement: “It is quite useless to argue the questions concerning the development of intimal sclerosis if we study and discuss the late stages of the disease alone … If we wish to gain a true insight into the complex question of arteriosclerosis, we must attempt to follow the lesion from its earliest beginning” [1]. Also aging is that, even at a very early age, a “normal” patient with an aorta completely free of structural alterations does not exist, i.e., the population cannot be divided into a group that has atherosclerosis and another group that does not [2]. Rather, the contiguous nature of the histologic changes and the time of life at which a specific change predominates indicate that each represents a gradation or stage in a temporal sequence (see below). The mechanisms involved in this long process are not entirely clear. Once symptom-producing lesions are present, lesion composition and architecture are complex and pathogenetic mechanisms are difficult to unravel. Atherogenesis can be better understood, and more successfully influenced, before lesions become clinically overt. Nevertheless, the definition of such initial atherosclerotic lesions inevitably is the result of a classification of the whole spectrum of atherosclerotic lesions from early to late stages (Table 1).

## 2. The Evolution of Classification Systems

Previous work considered the “simple” fatty streak the earliest lesion of atherosclerosis [2]. A fully developed classification was first provided by the AHA’s Committee on Vascular Lesions [42,43,44], later on updated [3] and modified [5], respectively. However, a shortcoming of the latter classification systems is that extracellular lipoprotein deposition as the prerequisite of foam cell formation is not considered. Unlike, extracellular lipoprotein deposition was considered in the elegant study of Nakashima et al. [8] on early human coronary atherosclerosis recognizing that fatty streaks develop via extracellular deposition of lipids associated with specific types of proteoglycans in the outer layer of preexisting diffuse or “nonatherosclerotic” intimal thickening (DIT) [5,8]. DIT was strongly expressed from an early age in arteries that are considered to be prone to atherosclerosis. These findings suggest that the development of atherosclerosis depends at least partly on the degree of DIT [45].

## 3. The Matter of Insudation

Previous observations suggested that plasma albumin and apolipoprotein B insudation is the earliest sign of a focal intimal lesion [9] and even before interstitial lipid deposits were discussed to result from the encrustation or imbibition of fibrin onto or into the intima [10].

Spontaneous insudation of LDL was demonstrated some 40 years ago [46,47]. The latter led to the “response to retention” hypothesis, which states that atherosclerosis develops in response to LDL entrapment [14,48]. This entrapment leads to a remarkable accumulation of LDL [49]. In contrast to other peripheral tissues, in which the concentration of LDL particles in the extracellular fluid is only 10% of that in the circulating blood, in the arterial intima the concentration of LDL-particles was found to be the same as in the circulation, i.e., it is 10-fold higher than in other tissues [11]. The observation that the extracellular fluid in the normal intima and the early developing lesions is not drained by lymphatic capillaries [13] might promote this concentration gradient by virtue of the absence of a potential system for removing protein, fluid and lipids from the arterial wall.

Atherosclerosis research typically focuses on the evolution of intermediate or advanced atherosclerotic lesions rather than on prelesional stages of atherogenesis. Yet these early events may provide decisive leads on the triggers of the pathologic process [15]. For example, apolipoprotein B insudation without macrophages as observed in our previous study was proposed to represent a prelesional stage that has hitherto been neglected and is not contained in current classifications of atherosclerotic lesion development [16]. In line with our results, a study on 38 Japanese autopsied subjects who died between 7 and 49 years of age reported extracellular deposition of lipids associated with specific types of proteoglycans before macrophage infiltration [8]. Both studies may provide a key in settling the chicken-vs.-egg debate of atherosclerotic plaque progression: does lipid come first, or do macrophages? 

## 4. Extra- or Intracellular Lipoproteins?

**Figure 1:**
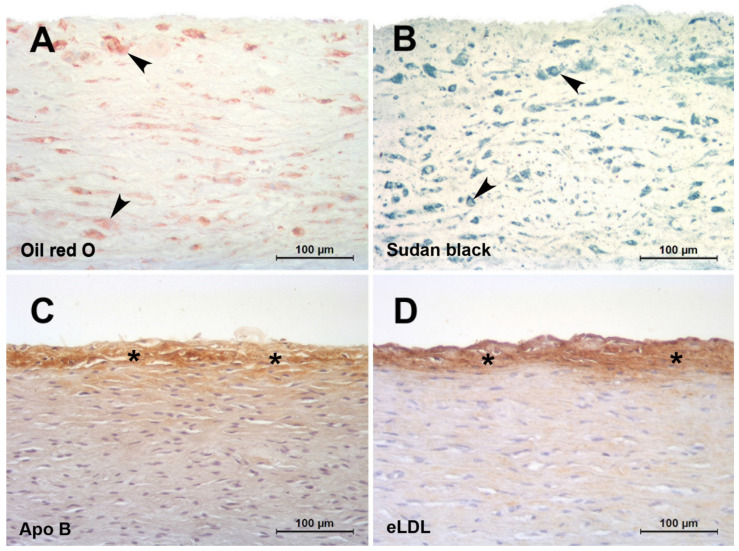
**Red herrings and hallmarks of the initial atherosclerotic lesion.** (**A**,**B**): Initial human aortic atherosclerotic lesions fixed in 4% formaldehyde and infiltrated and embedded in the water soluble plastic Technovit 7100 based on HEMA and GMA [53,54]. The use of alcohol containing fluids was strictly avoided. Staining with lipid-soluble dyes such as oil red O (**A**) or Sudan black (**B**) do not detect extracellular but only intracellular lipids (arrowheads). (**C**,**D**): Initial human aortic atherosclerotic lesions fixed in 4 % formaldehyde and infiltrated and embedded in paraffin. Staining with a polyclonal antibody against apolipoprotein B (**C**) [16] or AIL-3 (IgG_1_) against eLDL (**D**) [23]. Even after lipid extraction by the dehydration process, extensive extracellular epitopes (asterisks) can be detected in spite of the almost complete absence of macrophages.

In the classical paper by Geer et al., it was discussed that it is difficult to believe that initial accumulation occurs by phagocytosis of lipid filtered from the blood [26]. It was further stated that the existence of lipid-containing cells with cytoplasmic changes suggesting degeneration provides a possible explanation for the source of extracellular lipid. Moreover, and forward-looking, substantiation of this hypothesis was declared to be impossible until methods can be developed for the positive identification of extracellular lipid [26]. Indeed, lipid-soluble dyes such as Oil red O or Sudan black do not detect extracellular but only intracellular lipids (Figure 1A,B) putting across the latter untenable hypothesis. Not until elaborated staining methods like filipin or immunohistochemical staining (Figure 1C,D) revealed that extracellular particles constituted a significant component of accumulated cholesterol [50]. Accordingly, atherosclerosis is distinguished by the accumulation of lipoproteins within the arterial wall. An ionic interaction of positively charged regions of apolipoprotein B with matrix proteins, including proteoglycans, collagen, and fibronectin, is thought to initiate this process. Proteoglycans are complex glycoproteins containing highly negatively charged carbohydrate chains. These proteins are abundant in atherosclerosis lesions, and they associate with apolipoprotein B-containing lipoproteins [27]. Transgenic mice expressing modified apolipoprotein B that binds poorly to proteoglycans show reduced atheroma development [51]. This highlights the fundamental importance of LDL entrapment for initiating lesion formation rather than any modification of the lipoprotein occurring prior to insudation. It is generally held that progression of atherosclerosis displays the characteristic features of a chronic inflammatory process [52]. Hence, there must be a link between insudation of native LDL and inflammation. Native LDL lacks inflammatory properties, and it follows that the lipoprotein must undergo biochemical alterations to become atherogenic.

## 5. The Fate of Tissue-Stranded LDL

What happens to tissue-stranded LDL? What kind of modification takes place? Among several other candidates, two different concepts of lipoprotein modification are propagated: the widespread oxidation hypothesis [55] and the less common eLDL hypothesis, which proposes that modification of LDL occurs through the action of ubiquitous hydrolytic enzymes (enzymatically modified LDL or eLDL) rather than oxidation [17,18]. Originally, enzymatic modification of LDL in vitro was performed by sequential treatment with trypsin, cholesterol esterase and neuraminidase [20], the former later on replaced by other proteases at will [56]. As for the latter enzyme, multiple evidence has been provided that desialylation of LDL causes uncontrolled accumulation of lipids within the artery wall [57]. Importantly, both desialylation and proteolysis can sensitize LDL particles to other modifications via a cascade of well-arranged changes starting already in the blood comprising hydrolysis of the core cholesteryl esters and further to cholesterol crystallization [58]. It is well known for a long time that early atherosclerotic lesions contain much free cholesterol [28,35,50,59].

In general, global quantification indicates presence of only small amounts of oxidized lipids [60]. OxLDL detected within early human lesions is localized mainly intracellularly [29,61]. Napoli et al. analyzed fatty streaks in human fetuses. Epitopes of oxLDL were found, but these were localized mainly within macrophages [29]. Calara et al. reported that a single injection of heterologous LDL resulted in its accumulation in the arterial wall, where it became oxidatively modified within hours. However, the lipoprotein was localized mainly intracellulary in SMCs [61]. But the question of localization is exactly of paramount importance, because an extracellular atherogenic modification of LDL is mandatory to cause efficient cellular uptake of LDL. The oxidation hypothesis itself claims that oxidation is a prerequisite for cellular uptake of LDL. Another shortcoming of the oxidation hypothesis is that it does not provide for the fact that extracellular cholesterol in early lesions is mainly unesterified and can form crystals [28,35,50,59].

## 6. Atherogenesis in Infants and Children?

Arteries of infants and children may provide a model to study mechanisms involved in lesion initiation in the absence of many confounding factors occurring in adults. Several partly very old histological studies on normal and pathological conditions predominantly in carotid and coronary arteries of infants and children have been performed [2,62,63,64,65,66,67,68]. With the exception of our own study (see below) only two studies have addressed the temporal and spatial sequence of LDL insudation, extracellular modification and intimal monocyte accumulation in very early stages of lesion formation in premature fetuses [29] and children aged 1–13 years [69]. However, these studies did not address CRP deposition, eLDL formation or complement activation, important issues that were addressed in our study investigating very early stages of lesion formation in human aortas of infants and children below 1 year of age and up to the age of 15 years [16]. Acting with caution regarding the overall scarcity of lesions, novel insights were obtained into the temporal and spatial sequence of LDL insudation, modification and intimal monocyte accumulation in the earliest detectable stages of atherogenesis. Hitherto undescribed possible prelesional stages of atherogenesis were established. They are characterized by ‘inert’ lipoprotein insudation without monocyte/macrophage infiltration, lipoprotein modification and complement activation in individuals <1 year of age (Figure 1C) and an incipient enzymatic lipoprotein modification in individuals between 6 and 15 years of age [16] (Figure 1D).

## 7. Lesion Progression Due to…?

From a morphological point of view, repeated suggestions have been made to address lesions that represent the link between foam cell accumulations and atheromas; six of 32 fatty streaks (19%) investigated by Guyton and Klemp contained cholesterol crystals or clefts, which were found in the musculoelastic (deep) layer of the intima or in the tunica media [30]. Their findings suggest that the lipid-rich core region does not originate primarily from the debris of dead foam cells in the superficial intima, but instead arises from lipids accumulating gradually in the extracellular matrix of the deep intima corresponding to Stary’s preatheroma lesion [70,71]. Stary himself, however, claimed that preatheroma lesions contain small pools of lipid droplets and dead cell remnants as a source of extracellular lipid in addition to macrophage foam cells [4]. The earliest feature of a morphological bridge to more advanced plaques (progressive atherosclerosis) as described by the AHA classification is PIT, which is characterized by extracellular lipid accumulation (lipid pools) that are rich in extracellular matrix proteoglycans [5,6,7].

The initial atherosclerotic lesion is reversible and essentially harmless. What tips the scales towards irreversible, advanced lesions, i.e., the point of no return from a mechanistic point of view? In pursuit of an answer, it is important to clearly distinguish between initiation and progression of atherosclerotic lesion development. A factor intimately involved in the advanced stages of atherosclerosis and its sequelae does not necessarily play an important role in the initiation of atherosclerosis. Vice versa, a factor intimately involved in atherogenesis might become less important in the course of lesion progression [18].

First of all, the concept that “whole plasma” crosses endothelium, and the steady state concentrations reflect rates of egress of the macromolecules, which in turn depend on molecular sieving was propagated by Smith in 1977 [11]. Next, investigators have stressed the influx-efflux aspects of lipid metabolism in the arterial wall [12]. As already mentioned above, the absence of lymphatics draining the epicardial coronary arteries was considered a factor predisposing to coronary atherosclerosis by virtue of the absence of a potential system for removing protein, fluid and lipids from the arterial wall [13]. If the amount of insudated LDL exceeds the recycling capacity of the normal intima, or, in other words, the capacity of the system is overburdened, this would lead to an imbalance between lipoprotein and cholesterol deposition and removal, with subsequent accumulation of extracellular LDL particles [17,18]. The macrophages that entered the arterial wall and consumed the retained and modified lipoproteins would then persist rather than simply leave, secreting a variety of molecules that accelerate lipoprotein retention, plaque instability, and clotting on rupture [14].

## 8. The eLDL Hypothesis (Figure 2)

**Figure 2:**
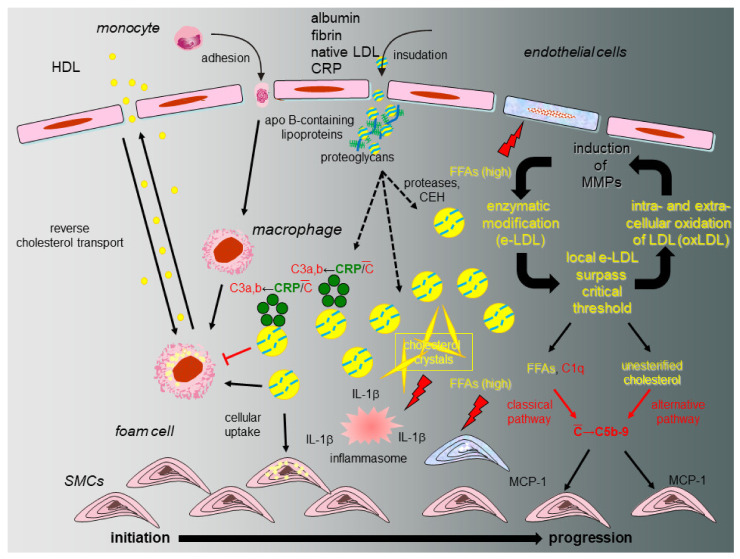
Proposed model of initiation and progression of atherosclerosis with special emphasis on the role of CRP and the complement system. Under normal circumstances (initiation and reversion, normocholesterolemia, left), native LDL entrapped within the arterial intima and associated with specific types of proteoglycans is enzymatically modified (eLDL), leading to a sequence of events that serve to clear the vessel wall of cholesterol. Binding of CRP to eLDL is the first trigger for complement activation (C), but in this early stage the terminal sequence is spared. The physiological sequence of events is concluded by reverse cholesterol transport. If the capacity of the system is overburdened (initiation and progression, hypercholesterolemia, right), this leads to accumulation of eLDL with subsequent generation of potentially harmful C5b-9 complexes by both the classical and alternative pathway as well as accumulation and oxidation of extracellular LDL particles followed by a wealth of well-documented events like MMP production in surrounding cells and subsequent amplification of enzymatic degradation of LDL. Hydrolysis of the core cholesteryl esters and subsequent cholesterol crystallization mediates formation of the inflammasome directly stimulating NLRP3 leading to increased tissue levels of IL-1 β. FFAs play multifaceted roles through their dual capacity to exert stimulatory and cytotoxic effects on neighboring cells (modified from [18]).

A fundamental question relates to the fate of tissue-stranded native LDL and the nature of the modification that bestows novel properties onto the lipoprotein. As already indicated above, during the last 25 years, we accumulated evidence in support of a different concept on atherogenesis [15,17,18] distinguishing between atherosclerotic lesion initiation with reversion or lesion initiation with progression (Figure 2). Our hypothesis heeds the above-mentioned shortcomings of the oxidation hypothesis and proposes that modification of native LDL occurs through the action of ubiquitous hydrolytic enzymes (enzymatically modified LDL or eLDL) rather than oxidation and contends that the physiological events leading to macrophage uptake and reverse transport of eLDL first occur without inflammation (initiation with reversion). Under hypercholesterolemic conditions, however, the cholesterol removal system is overburdened due to disequilibrium between rates of insudation and reverse transport followed by excessive tissue-stranding of eLDL. Unhalted activation of innate immune effectors, in particular complement and other detrimental effects follow [72]. Overburdening of the physiological eLDL removal machinery is further accompanied by IL-6 production possibly assuming a critical role [19] which, by the way, could explain the slightly elevated CRP levels. LDL additionally activates the alternative complement pathway [17,20] possibly due to the free cholesterol itself. The demonstration of C5b-9 in early human atherosclerotic lesions and its colocalization with SMCs [21], as well as the complement induced release of MCP-1 from human SMCs [22], provided further evidence of a possible role of complement activation in the early stages of atherogenesis. Notably, our work indicated that eLDL might activate complement in atherosclerotic lesions via CRP-dependent and -independent pathways and later on demonstrated that the CRP dependent pathway halts before the proinflammatory terminal sequence, while the CRP-independent pathway proceeds to completion with the generation of C5b-9 complexes [17,21,23,24,25].

In summary, evidence was accumulated in support of a different concept on atherogenesis (summarized in [15,17,18]) proposing that modification of native LDL occurs through the action of ubiquitous hydrolytic enzymes rather than oxidation and contending that the physiological events leading to macrophage uptake and reverse transport of eLDL first occur without inflammation. As already stated above and also by Holman et al. in 1958, the factors responsible for succeeding stages of atherosclerosis may be different from those which initiated the first stage. “Intelligent therapeutic attempts must take cognizance of these different stages, for that treatment which is effective in one stage may be ineffective or even contra-indicated in another stage” [2]. In other words, preventing or reversing initial atherosclerotic lesions would avoid the later stages and therefore prevent clinical manifestations. The response to retention hypothesis directly supports the strategy of lowering plasma levels of atherogenic lipoproteins as the most successful therapy for atherosclerosis and its sequelae [14].

## 9. Alternative Hypotheses on Atherosclerotic Lesion Initiation and Progression

Apart from but unquestionable closely related to the above-mentioned mechanistic considerations, several other hypotheses on atherosclerotic lesion initiation and progression merit consideration and discussion.

### 9.1. Vascular-Associated Lymphoid Tissue (VALT)

Wick’s group coined the term VALT for the accumulation of mononuclear cells at regions of the arterial wall in healthy children and adolescents that are predisposed to the development of atherosclerotic lesions later in life if risk factors are present. Accordingly, they claimed to demonstrate that immunological-inflammatory cells are present “in the earliest stages of atherogenesis” in 15–34 year old subjects, arguing in favor of an initiating role of the immune system in atherosclerosis development [31,32]. However, on closer examination fatty streaks are put on a level with “the earliest stages of atherogenesis”. Without challenging the importance of the immune system in atherosclerosis development, fatty streaks cannot be declared as earliest stages of atherogenesis.

### 9.2. Lysosomal Storage Disease

Following the hypothesis by de Duve et al. defining foam cell formation in atherosclerosis as a variant of lysosomal storage disease [33], Bobryshev et al. investigated parameters of the regulatory system of expression of genes relating to the lysosomal function of intimal cells residing in the initial lesion (Type I lesion) [34]. In addition to altered patterns of CD68 distribution, substantial structural changes of lysosomes in the ‘normal intima-initial lesion-fatty streak’ sequence were observed as well. These changes included the alteration in electron density of the matrix of lysosomal bodies as well as the formation of lamellar bodies in lysosomes which might reflect the summary of a variety of altered metabolic pathways rather than specific gene defects. It is suggested that the observed occurrence of the dysregulation in the expression of the lysosome-relating gene may contribute to the inability of lysosomes to cope with a high intake of lipids in developing fatty streaks [34].

### 9.3. Inflammasome and Mitochondria

Another approach relates to the concept of the inflammasome termed in 2002 describing multimolecular complexes formed in the cell cytosol upon stimulation [73]. The inflammasome is a complex of distinct proteins which, when brought together, serve to convert inactive IL-1 β to the active proinflammatory cytokine IL-1 β. The formation can be mediated by multiple different signals including cholesterol crystals and oxLDL deposited in vessel walls or mitochondrial DAMPs released from damaged cells (see below) and directly stimulating NLRP3 inflammasome leading to increased tissue levels of IL-1 β and inflammation [36]. Very interestingly, it was demonstrated that minute cholesterol crystals are present already in early diet-induced mice atherosclerotic lesions and that their appearance coincides with the first appearance of inflammatory cells suggesting that crystalline cholesterol acts as an endogenous danger signal and its deposition in arteries is an early cause rather than a late consequence of inflammation [36] (As a comprehensive appreciation of atherosclerotic lesion development in relevant animal models would go beyond the scope of the present review, the reader is referred to an excellent recent presentation of this issue [74]). This assumption is in perfect accordance with the proposed significance of unesterified cholesterol in early human atherosclerotic lesions (see above). Provided that the crystalline form of cholesterol may be mandatory in driving arterial inflammation, this may offer new therapeutic targets, e.g., solubilization of cholesterol crystals and anti-IL-1 β treatment strategies on the one hand [75] and explain the apparent paradox that ACAT inhibitors tested in large clinical trials showed not a decrease but rather an increase in the size of the coronary atheroma [76,77] on the other hand, because the crystalline form of cholesterol would be expected to be increased after inhibition of ACAT. 

Mitochondria are another possible candidate for initiation and regulation of NLRP3 (see above). On the one hand, mitochondrial DAMPs are released from damaged cells contributing to the stimulation and/or perpetuating of innate immunity. On the other hand, the presence of certain mtDNA mutations leading to defective mitophagy can prevent the arrest of a proinflammatory response or even increase it upon repeated stimulation [78].

### 9.4. Micro RNAs

Accumulating evidence indicates that numerous miRNAs play a pivotal role in both atherosclerotic initiation and progression. miRNAs are highly conserved, single-stranded noncoding RNA molecules, exerting post-transcriptional effects on gene expression by promoting degradation of mRNA target and/or inhibiting mRNA translation [37]. Accordingly, they participate in positive as well as negative regulatory loops resulting in a hierarchical network. From both in vitro and in vivo studies, it is evident that miRNAs regulate diverse cell types involved in atherosclerotic disease initiation and progression. They mediate cellular regulation in endothelial activation and inflammation [38], regulate the differentiation of macrophages and their polarization [39], which is a critical component of the inflammatory response and play a central role in the mechanisms determining SMC phenotype [40]. Moreover, circulating miRNAs are promising new candidates as biomarkers for atherosclerosis and pharmacological targeting of dysregulated miRNAs seems to be a promising therapeutic concept [41,79].

## 10. Conclusions

Direct anti-inflammatory therapies may prove successful as adjuvant components but will likely never be used in the absence of strategies to lower plasma levels of atherogenic lipoproteins, the key point of the perception that atherosclerosis is not simply an inevitable result of senescence [2]. In particular, given the importance of chemical modifications for lipoprotein atherogenicity, regulation of the enzymes involved might be a tempting target for pharmacological research [57].

## Figures and Tables

**Table 1:** **Concepts on initiation and/or progression of human atherosclerotic lesion.** Denomination: terminology used to describe the beginning of atherosclerotic lesion development. Features: hallmarks and triggers of lesion initiation. Lesion progression due to…: hallmarks and triggers of lesion progression (provided that lesion progression is considered).

Denomination	Features	Lesion Progression Due to …	Ref.
fatty streak, minimal sudanophilic intimal deposit	both intra- and extracellular “globules” of lipid, slight increase in interstitial mucinous material	conversion into fibrous plaques	[2]
type I (initial) lesion	isolated macrophage foam cells	small pools of lipid droplets and dead cell remnants as a source of extracellular lipid in addition to macrophage foam cells (preatheroma)	[3,4]
intimal xanthoma	isolated macrophage foam cells	extracellular lipid accumulation (lipid pools) that are rich in extracellular matrix proteoglycans (pathologic intimal thickening (PIT))	[5,6,7]
grade of lipid deposition 1	fatty streaks with extracellular lipids colocalizing with biglycan and decorin in the outer layer of the intima	n/a	[8]
early lesion	plasma albumin and apolipoprotein B insudation	n/a	[9]
early lesion	interstitial lipid deposits resulting from the encrustation or imbibition of fibrin onto or into the intima	n/a	[10]
gelatinous lesion	balances of intact LDL/“deposited” cholesterol and of fibrinogen/fibrin	loss of steady state concentrations reflecting rates of egress of macromolecules depending on molecular sieving (immobilization of LDL by fibrin)	[11]
n/a	n/a	influx-efflux imbalance in the cell and blood vessel wall	[12]
epicardial coronary atherosclerosis	impairment of lymphatic drainage from the coronary arteries (absence of a potential system for removing protein, fluid and lipids from the arterial wall)	impairment of lymphatic drainage from the coronary arteries (absence of a potential system for removing protein, fluid and lipids from the arterial wall)	[13]
prelesional stage	‘inert’ lipoprotein insudation without monocyte/ macrophage infiltration, lipoprotein modification and complement activation	overload of the cholesterol removal machinery, enzymatic modification of LDL, complement activation, persisting macrophages secreting a variety of molecules accelerating lipoprotein retention, plaque instability, and clotting on rupture	[14,15,16,17,18,19,20,21,22,23,24,25]
early fatty streak	intracellular lipid accumulation in SMCs	degeneration of lipid-containing cells with extravasation of lipid particles into the extracellular space	[26]
early lesion	ionic interaction of positively charged regions of apolipoprotein B with matrix proteins, including proteoglycans, collagen, and fibronectin	n/a	[27]
initial lipid deposition	unesterified cholesterol-rich lipid particles	n/a	[28]
fatty streak	LDL accumulation and oxidation preceding intimal accumulation of monocytes	n/a	[29]
n/a	n/a	cholesterol crystals or clefts in the musculoelastic (deep) layer of the intima or in the tunica media	[30]
fatty streak	accumulation of mononuclear cells	n/a	[31,32]
type I (initial) lesion	alteration in electron density of the matrix of lysosomal bodies as well as the formation of lamellar bodies in lysosomes	substantial structural changes of lysosomes in the ‘normal intima-initial lesion-fatty streak’ sequence	[33,34]
early lesion	unesterified, crystalline cholesterol	n/a	[35,36]
initial lesion	miRNAs mediating cellular regulation in endothelial activation and inflammation, differentiation of macrophages and their polarization, having important functional properties in lipoprotein homeostasis and playing a central role in the mechanisms determining SMC phenotype	miRNAs mediating cellular regulation in endothelial activation and inflammation, differentiation of macrophages and their polarization, having important functional properties in lipoprotein homeostasis and playing a central role in the mechanisms determining SMC phenotype	[37,38,39,40,41]

## Abbreviations

ACAT	acetyl-CoA acetyltransferase
AHA	American Heart Association
CRP	C-reactive protein
DAMPs	damage-associated molecular patterns
DIT	diffuse intimal thickening
eLDL	enzymatically modified LDL
FFA	free fatty acid
GMA	glycol methacrylate)
HEMA	2-hydroxyethylmethacrylate
IL-1 β	interleukin-1 β
IL-6	interleukin-6
LDL	Low Density Lipoproteins
MCP-1	monocyte chemotactic protein-1
mtDNA	mitochondral DNA
miRNAs	micro RNAs
MMP	matrix metalloproteinase
NLRP3	NOD-, LRR- and pyrin-domain containing 3
oxLDL	oxidized LDL
PIT	pathological intimal thickening
SMC	smooth muscle cell
VALT	vascular-associated lymphoid tissue
